# Analysis of Molecular Heterogeneity in Therapeutic IFNα2b from Different Manufacturers by LC/Q-TOF

**DOI:** 10.3390/molecules25173965

**Published:** 2020-08-31

**Authors:** Lei Yu, Lei Tao, Yinghua Zhao, Yonghong Li, Dening Pei, Chunming Rao

**Affiliations:** 1 Division of Recombinant Drugs, National Institute for Food and Drug Control (NIFDC), Tiantan Xili No. 2, Dongcheng District, Beijing 100050, China; yulei@nifdc.org.cn (L.Y.); taolei01@nifdc.org.cn (L.T.); liyh@nifdc.org.cn (Y.L.); 2 SCIEX China, Beijing 100015, China; yinghua.zhao@sciex.com

**Keywords:** interferon α2b variants, LC/Q-TOF, molecular heterogeneity, N-terminus, oxidation, acetylation, deamidation

## Abstract

Recombinant human IFNα2b (rhIFNα2b), as an important immune-related protein, has been widely used in clinic for decades. It is also at the forefront of the recent emergence of biosimilar medicines, with numerous products now available worldwide. Although with the same amino acid sequence, recombinant proteins are generally heterogeneous due to post-translational modification and chemical reactions during expression, purification, and long-term storage, which could have significant impact on the final product quality. So therapeutic rhIFNα2b must be closely monitored to ensure consistency, safety, and efficacy. In this study, we compared seven rhIFNα2b preparations from six manufacturers in China and one in America, as well as four batches of rhIFNα2b preparations from the same manufacturer, measuring IFNα2b variants and site-specific modifications using a developed LC/Q-TOF approach. Three main forms of N-terminus, cysteine, methionine, and acetylated cysteine were detected in five rhIFNα2b preparations produced in *E. coli* (1E~5E) and one in *Pseudomonas* (6P), but only the native form with N-terminal cysteine was found in rhIFNα2b preparation produced in *Saccharomyces cerevisiae* (7Y). Two samples with the lowest purity (4E and 6P), showed the highest level of acetylation at N-terminal cysteine and oxidation at methionine. The level of oxidation and deamidation varied not only between samples from different manufacturers but also between different batches of the same manufacturer. Although variable between samples from different manufacturers, the constitution of N-terminus and disulfide bonds was relatively stable between different batches, which may be a potential indicator for batch consistency. These findings provide a valid reference for the stability evaluation of the production process and final products.

## 1. Introduction

Among the interferons (IFNs), IFN-α2 has been the most broadly evaluated clinically. IFN-α2 is an important immune-related protein, which has multiple functions in antiviral process, antiangiogenic, antiproliferative, and proapoptotic effects in cancers. The U.S. Food and Drug Administration approved human IFN-α2a and IFN-α2b in 1986 for the treatment of hairy cell leukemia [1,2,3]. Recombinant human IFN α2b is a 165-amino acid single chain polypeptide with a molecular weight 19.26 kDa (Chinese Pharmacopoeia, 2020 version). Recently commercial IFNα2b can be produced in *Escherichia coli (E. coli)*, *Pseudomonas*, yeast or mammalian cells [4,5].

Recombinant proteins are generally heterogeneous due to post-translational modification and chemical reactions (such as oxidation, deamidation, aggregation, and so on) during expression, purification, and long-term storage [6]. Heterogeneity is mainly reflected in the difference of charge, hydrophobicity, change of molecular weight, different type of disulfide bonds, etc. [7,8]. Endogenous proteins are structurally heterogeneous but still recognized as self by the immune system; however, recombinant proteins produced in heterologous systems may include structural variants that are non-self and potentially immunogenic [9]. Protein variants can be classified as either product-related impurities that have a negative impact on safety and efficacy or product-related substances that have no negative impact on safety and efficacy [7]. Downstream processing, which includes various chromatography and filtration steps, is optimized to remove process and product related impurities. However, these steps will also have a significant impact on the nature and levels of variants at drug substance levels. It is worth noting that targeting to remove all the heterogeneity through downstream processing is not practical. Therefore, modifications and degradations that inevitably occur during the manufacturing process have a significant impact on the final product quality [7].

The detection of product variant is pivotal for quality control. Besides, monitoring the type or level of modification is expected to reflect the stabilization of production process and consistency between different batches of products. Therefore, state-of-the-art physiochemical and analytical methods as well as biological assays are required to characterize the biological products in full for therapeutic applications. To monitor the purity and the biochemical and biological properties of rhIFNα2b, appropriate molecular and biological characterization methods should be developed [10].

Mass spectrometry (MS) has the advantages of high precision and high resolution in identifying structural and molecular information of micro-sample. MS can deliver the measurement of intact molecular weight, and in combination with peptide mapping it also can identify sequence variation and site modification. High resolution MS such as Quadrupole Time-of-Fight (Q-TOF) are excellent analytical tools for assessing protein heterogeneity and for structural characterization, and high-resolution techniques such as Ultra Performance Liquid Chromatography (UPLC) have been successfully coupled to high resolution MS analyzers in cases where the sample is complex and highly heterogeneous [6,11].

In this study, seven recombinant human IFNα2b preparations from six manufacturers in China and one in America were analyzed and compared by a developed LC/Q-TOF method. IFNα2b variants and site-specific modifications were identified and quantified both in intact protein level and peptide level.

## 2. Results

### 2.1. Intact Protein Analysis

#### 2.1.1. Purity Analysis

**Figure 1:**
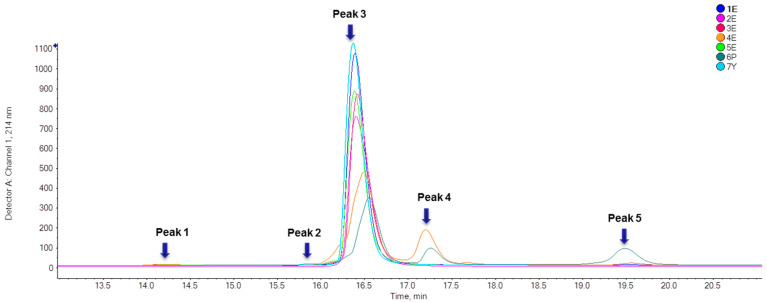
UV chromatograms of seven IFNα2b preparations. 1E~5E were expressed in *E. coli*, 6P was expressed in *Pseudomonas* and 7Y was expressed in *Saccharomyces cerevisiae*.

Seven IFNα2b preparations were analyzed by a developed LC/Q-TOF to reveal the molecular heterogeneity at intact protein level. The chromatograms of UV detector at 214 nm were shown in Figure 1. As listed in Table 1, the area percentages of the main peak at about 16.4 min based on UV signal were above 99.0% except for 4E (74.9%) and 6P (66.0%).

#### 2.1.2. Identification of IFNα2b Variants

**Figure 2:**
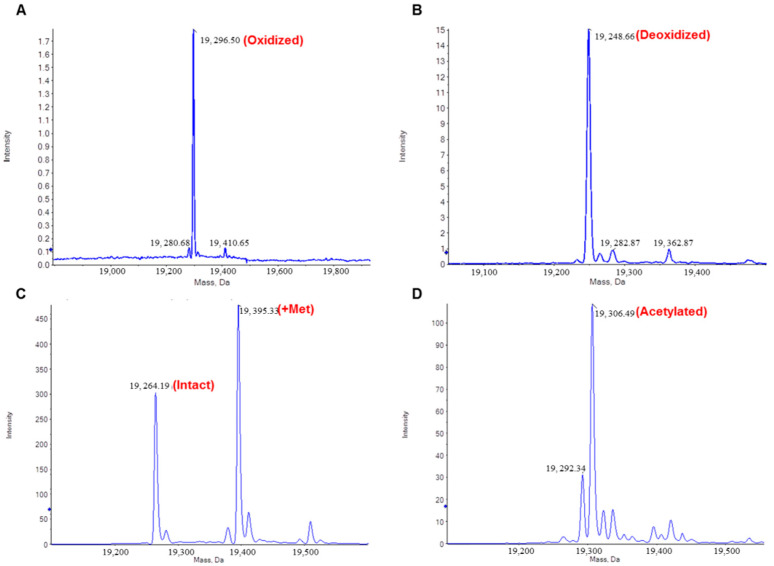
Identification of IFNα2b variants based on deconvoluted mass spectra. (**A**). Oxidized IFNα2b in peak 1. Spectrum from 14.095 to 14.420 min of sample 6P. (**B**). Deoxidized IFNα2b in peak 2. Spectrum from 15.927 to 16.098 min of sample 7Y. (**C**). Native and +Met IFNα2b in peak 3. Spectrum from 16.509 to 16.920 min of sample 6P. (**D**). Acetylated IFNα2b in peak 4. Spectrum from 17.246 to 17.588 min of sample 6P. Input spectrum isotope resolution: 3,000.

IFNα2b variants were identified based on MS molecular weight (MW). The deconvoluted mass spectra were shown in Figure 2 and non-convoluted mass spcetra refer to Supplementary Figure S1. The native IFNα2b molecule contains 165 amino acid residues (CDLPQTHSLGSRRTLMLLAQMRRISLFSCLKDRHDFGFPQEEFGNQFQKAETIPVLHEMIQQIFNLFSTKDSSAAWDETLLDKFYTELYQQLNDLEACVIQGVGVTETPLMKEDSILAVRKYFQRITLYLKEKKYSPCAWEVVRAEIMRSFSLSTNLQESLRSKE) and two disulfide bonds, one between Cys 1 and Cys 98 and another between Cys 29 and Cys 138, according to Chinese Pharmacopeia, 2020 version. The theoretical molecular weight is 19,264.91 Da. As shown in Figure 2C, the native form mainly appeared in peak 3 at about 16.4 min, and a “+Met” form, the direct translation product of the gene retaining the N-terminal Met, was also found in peak 3, suggesting the addition of N-terminal Met has no obvious influence with the retention time of IFNα2b (Figure 2C). Except for “+Met”, other modified forms of IFNα2b were also identified: N-terminal acetylated (increase of 42.04 Da), oxidized (increase of about 16.00 Da at one site and 32.00 Da at two sites), and deoxidized (decrease of 16.00 Da), and all of the errors were below 1 Da. The theoretical and measured MWs of each form were listed in Table 2. Only a trace amount of deoxidized form was found in sample 7Y, but the oxidized form was found in sample 4E and 6P, and the proportion was about 24.2% for 4E and 14.7% for 6P (Table 3), which may be a cause for the poor purity.

The variants with an increase of about 1 Da and 2 Da were found, suggesting deamidation and free thiols might exist. But the data accuracy was insufficient to properly distinguish these variants with small difference at MW. Besides, although a small quantity of dimer forms were found in sample 6P, the measured molecular weight (about 38.5 KDa) was highly variable, which could be caused by multiple modifications.

#### 2.1.3. N-Terminal Heterogeneity

**Figure 3:**
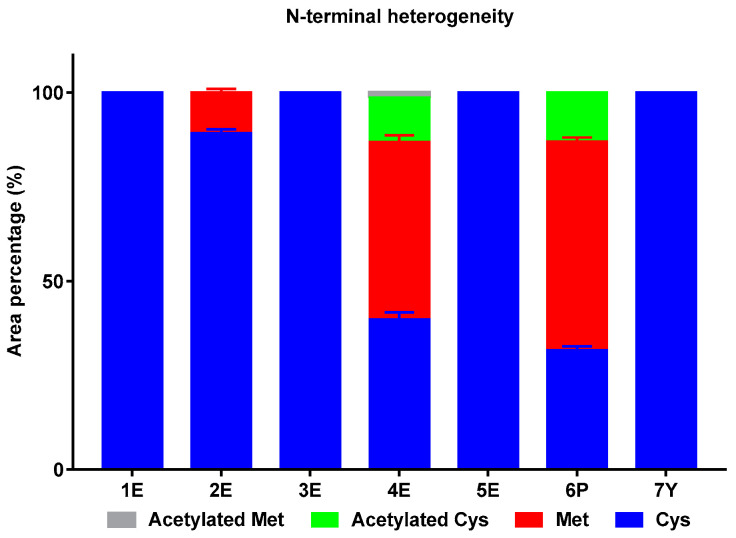
Percentage of IFNα2b variants with different N-terminus.

Four forms of N-terminus were found: Met, Cys, acetylated Cys, and acetylated Met, and the percentage of each form were shown in Figure 3. For samples 1E, 3E, 5E, and 7Y, only native form with N-terminal Cys was found. For sample 2E, about 10% has an addition of N-terminal Met. For sample 4E and 6P, a large proportion of N-terminal Met and acetylated Cys existed, even more than native form, which may be another cause of poor purity. Moreover, a small amount of acetylated-Met was found in sample 4E (about 1.3%).

### 2.2. Evaluation of Modifications in IFNα2b by Peptide Mapping

#### 2.2.1. Peptide Map

**Figure 4:**
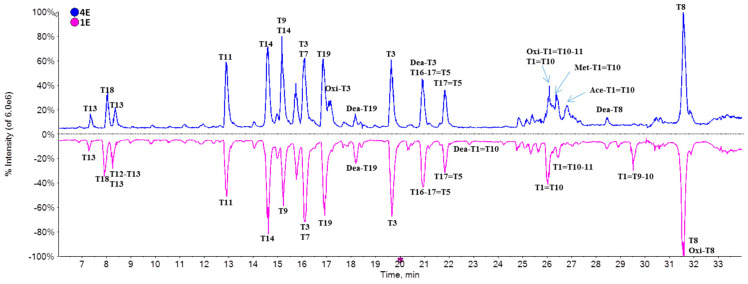
Peptide maps of sample 1E and 4E.

To further study the molecular heterogeneity, the IFNα2b samples were digested by trypsin and analyzed at peptide level. The distribution and intensity of peaks in the peptide maps of seven samples were basically consistent, except for partial regions, and amino acid sequences coverage was above 99%. Taking sample 4E for example, the peaks between 26 min and 27.5 min were notably different from sample 1E, for substantial “+Met” and acetylated IFNα2b variants existed in sample 4E (Figure 4).

#### 2.2.2. Modifications in IFNα2b from Different Manufacturers

Modifications at potential sites were next analyzed at peptide level, including remain of N-terminal Met, N-terminal acetylation, fracture or mismatch of disulfide bond, oxidation at Met, and deamidation at asparagine (Asp), which are incidental in recombination proteins. The results showed that the level of each modification significantly varied among different samples.

**Figure 5:**
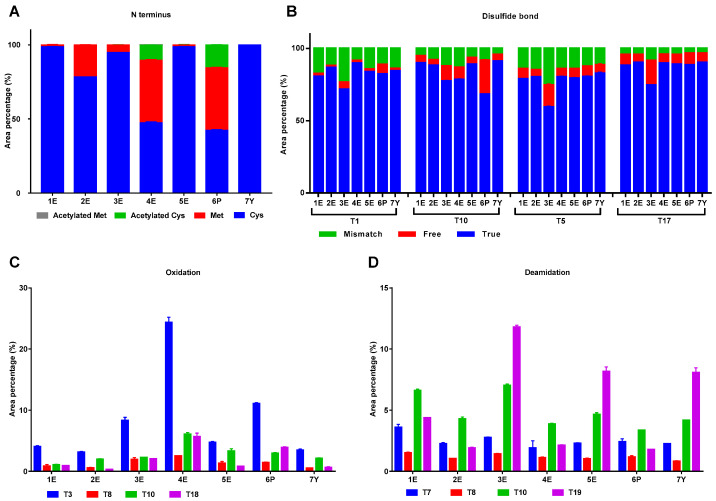
Modifications in IFNα2b from different manufacturers. (**A**). N-terminal amino acid residue; (**B**). Disulfide bonds; (**C**). Oxidation in peptides containing Met residue including T3, T8, T10 and T18; (**D**). Deamidation in peptides containing Asp residue including T7, T8, T10 and T19.

Four forms of N-terminus were screened according to the results of intact protein analysis: Met, Cys, acetylated Cys and acetylated Met. As shown in Figure 5A, no Met was found in N-terminal of IFNα2b preparation produced in *Saccharomyces cerevisiae* (sample 7Y), and the highest level of N-terminal Met (about 37%) was found in IFNα2b preparation produced in *Pseudomonas* (sample 6P). The proportion of N-terminal Met varied between different IFNα2b preparations produced in *E. coli* expression system: sample 4E was high (35%), sample 2E was moderate (16%), sample 3E was low (3%) and sample 1E and 5E were extremely low (below 1%). Acetylated Cys was found in sample 4E and 6P, and only a little amount of acetylated Met was found in sample 4E (about 0.16%). These results were basically consistent with the results of intact protein analysis.

Two disulfide bonds were formed in native IFNα2b, with one between T1 (Cys1) and T10 (Cys 98) and another between T5 (Cys 29) and T17 (Cys 138). To evaluate the disulfide bonds, we analyzed four peptides containing Cys, searched “true” forms with correct disulfide bonds (T1 = T10 and T5 = T17), “free” forms with free thiols, and “mismatch” forms with incorrect disulfide bonds (T1 = T5, T1 = T17, T5 = T10 and T10 = T17) and calculated the percentages of each form of peptides. The results were shown in Figure 5B. The percentage of correct disulfide bonds was the lowest in sample 3E.

Oxidation always happens in Met residue, so the level of oxidation in four peptides containing Met residue, T3 (Met 16 & Met 21), T8 (Met 59), T10 (Met 111), and T18 (Met 148) were analyzed and calculated. As shown in Figure 5C, the oxidation level of sample 4E was the highest followed by sample 6P, which is consistent with the results of intact protein analysis. T3 was the most oxidized peptide for it has two Met residues.

Another highly frequent chemical modifications in proteins is the deamidation of Asp residue. Four peptides containing Asp residue, T7 (Asp 45), T8 (Asp 65), T10 (Asp 93), and T19 (Asp 156) were assessed. As shown in Figure 5D, Asp 156 was the most deamidated site, and Asp 65 was the least deamidated site.

#### 2.2.3. Modifications in IFNα2b of Different Batches

**Figure 6:**
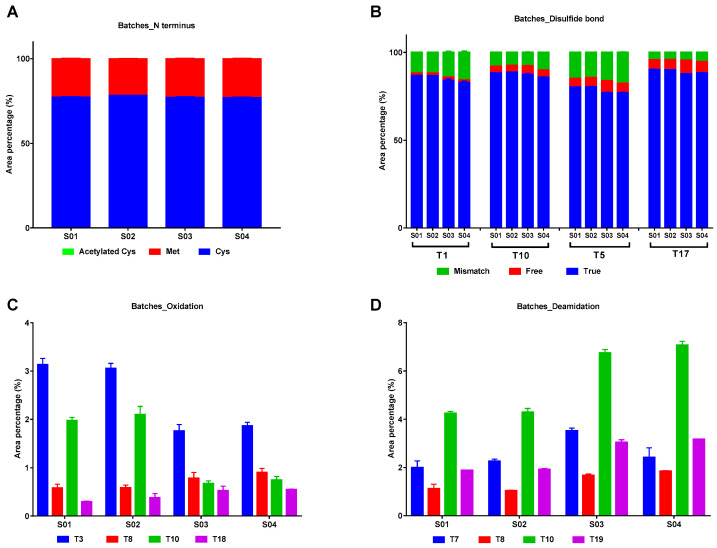
Modification in IFNα2b of different batches from the same manufacturer. (**A**). N-terminal amino acid residue; (**B**). Disulfide bonds; (**C**). Oxidation in peptides containing Met residue including T3, T8, T10 and T18; (**D**). Deamidation in peptides containing Asp residue including T7, T8, T10 and T19.

Four batches of IFNα2b preparations from the same manufacturer were analyzed to evaluate the batch-to-batch consistency in modification. The constitution of N-terminus, disulfide bonds, oxidation and deamidation were shown in Figure 6. No significant difference was found in the constitution of N terminus among different batches (Figure 6A). The proportion of “mismatch” and “free” disulfide bonds in S01 and S02 was a little higher than S03 and S04 (Figure 6B). As to the oxidation rate, T3 and T10 in S01 and S02 were higher than S03 and S04, while the image is the reverse for T8 and T18 (Figure 6C). The overall deamidation rate in S01 and S02 were lower than S03 and S04 (Figure 6D).

## 3. Discussion

Protein synthesis is always initiated with either Met or *N*-formyl Met. For most proteins, the N-terminal Met residue is removed enzymatically after the initiation of translation, which is often crucial for the function and stability of proteins [12,13,14]. In *E. coli*, this step is controlled by the methionyl aminopeptidase (MetAP) [13]. If the next residue in the sequence has a small side chain such as Cys, just like IFNα2b, the removal of N-terminal Met is more likely to happen [15,16]. However, the N-terminal Met can never be completely removed. Consequently, this modification can still be observed in substantial amounts in mature IFNα2b obtained from *E. coli* expression systems [17], which was further confirmed by this study. N-terminal Met residue was detected in samples 1E~5E expressed in *E. coli* and 6P expressed in *Pseudomonas* at peptide level, although not detected in sample 1E, 3E, and 5E at intact protein level. However, no N-terminal Met could be detected in sample 7Y expressed in *Saccharomyces cerevisiae*, suggesting that a more rigorous mechanism to remove the N-terminal Met existed in eukaryotic cells. The removal of Met had no effect on the retention time, so two forms co-existed in peak 3(Figure 1). Current methods for purity analysis such as RP-HPLC and SEC-HPLC hardly separate “+Met” from native IFNα2b, which necessitates the use of high-resolution MS methods.

In eukaryotic cells, up to 98% proteins are N-terminally acetylated, and acetylation could stabilize the protein by protecting it from degradation via N-terminal proteases [18]. Although *N*-terminal acetylation was considered to be infrequent in prokaryotic cell [18,19], in this case acetylation was found in samples 4E and 6P, which was consistent with a previous report [17], suggesting that N-terminal acetylation may occur in recombinant proteins from bacteria expression systems. It was reported that acetylation could occur at N-terminal Cys residue in IFNα2b, and the acetylated form had only 10% of the activity of native molecule [17]. To overcome this N-terminal heterogeneity, many strategies have been developed, such as engineered interferon derivatives with phenylalanine residue directly downstream of the N-terminal Met [20], and co-expression of engineered MetAP in *E. coli* [14].

The formation of disulfide bonds is necessary to the spatial structure of proteins. We observed over 10% of “free” and “mismatch” forms in all samples including five produced in *E.coli*, one in *Pseudomonas* and one in *Saccharomyces cerevisiae*. The percentages of correct forms varied between different manufacturers, and relatively stable between different batches, demonstrating the status of disulfide bonds were related to expression procedure. Oxidation of Met residues and deamidation of Asp residues are two of the most common forms of chemical modifications that compromise the efficacy of therapeutic proteins [21]. In intact protein analysis, oxidized IFNα2b was only found in sample 4E (24.2%) and 6P (14.7%), but in peptide mapping, oxidized peptides were found in all seven samples, suggesting that oxidation could happen during sample preparation. Moreover, lesser sensitivity and multisite modifications could also be reasons for fewer variants were found in intact protein analysis. These data indicate that although inevitable, these chemical modifications could be restricted by optimizing the manufacturing process and storage conditions.

## 4. Materials and Methods

### 4.1. Materials

Iodoacetamide (IAA), guanidine hydrochloride, ammonium bicarbonate (NH_4_HCO_3_), and trifluoroacetic acid (TFA) were obtained from Sigma-Aldrich (St. Louis, MO, USA), acetonitrile (ACN) and water were from Fisher Scientific (Ottawa, Canada). All reagents were of analytical grade. Trypsin was purchased from Promega (Madison, WI, USA), and 3kD ultrafiltration device was from Millipore (Tullagreen, Ireland).

Recombinant human IFNα2b preparations 1E~5E, 6P, and 7Y were archived samples that had been preserved at −80 °C in National Institute for Food and Drug Control (NIFDC), China, provided by six manufacturers from China and one manufacturer from America. Samples were anonymised and identified via randomly assigned codes 1–7. Samples 1E~5E were manufactured in *E. coli* expression system, 6P was manufactured in *Pseudomonas* expression system and 7Y was manufactured in *Saccharomyces cerevisiae* expression system. Sample 2E_1~4 were four batches of 2E from the same manufacturer. The production dates of these samples ranged between 2017 and 2018, and the analyses were conducted in 2019.

### 4.2. Sample Preparation

For intact protein analysis, all samples were directly diluted to 5 mg/mL in solvent A (98% water, 2% ACN, 0.1% TFA) for LC/MS analysis.

For peptide mapping, 100 μg of proteins in each sample were denatured in 6 M guanidine hydrochloride solution, and then alkylated with 100 mM IAA in the dark for 30 min. The solution was centrifuged at 14,000× *g* through 3 kD filters to remove the guanidine hydrochloride, and the intercepted proteins were rinsed twice with 50 mM NH_4_HCO_3_. Protein digestion was done by adding 2 µg of trypsin to each sample and followed by incubation for 15 h at 37 °C. Finally, the peptides were centrifuged at 14,000× *g* for 15 min through 3 kD filters and the filtrate was adjusted to a concentration of 1 μg/μL with solvent A.

### 4.3. LC-MS/MS

The intact protein and peptide mapping samples were all analyzed with an UPLC, ExionLC coupled with a Q-TOF MS system, X500B (SCIEX, Framingham, MA, USA). The Photo-Diode Array (PDA) detector was integrated into the UPLC system, the wave length was set to 214 nm. The mobile phase consisted of solvent A (98% water, 2% ACN, 0.1% TFA) and solvent B (98% ACN, 2% water, 0.1% TFA). The wavelength of UV detector was set to 214 nm. SCIEX OS 1.4 software (SCIEX, Framingham, MA, USA) was used for control of data acquisition.

For intact protein analysis, the proteins were loaded on a BEH C18 column (1.7 μm, 2.1 mm × 100 mm, Waters, Milford, MA, USA) and separated with a 35 min gradient from 35–60% solvent B over 20 min. The flow rate was set to 0.2 mL/min. For MS acquisition, positive ion mode was applied. The spray voltage was set to 4500 V, the declustering potential (DP) was 145 V, the curtain gas was 30 psi, and the heated interface was set to 500 °C. The mass range *m*/*z* was set to 600~5000 with a 1 s accumulation time.

For peptide mapping analysis, the peptides were separated with a BEH C18 column (1.7 μm, 2.1 mm × 100 mm, Waters, Milford, MA, USA) and a 45 min gradient from 5–55% solvent B. The flow rate was 0.2 mL/min. For MS acquisition, the spray voltage was set to 5500 V, DP was 100 V, the curtain gas was 30 psi, and the heated interface was set to 550 °C. The information dependent acquisition (IDA) mode was applied. For TOF MS scan, positive ion mode was applied, the mass range *m*/*z* was set to 200~2,000 with a 250 ms accumulation time. For TOF MS/MS scan, 15 candidate ions per TOF MS scan were selected for analysis, dynamic collision energy was applied. The mass range *m*/*z* was set to 100~2,000 with a 50 ms accumulation time.

SCIEX OS 1.4 software (SCIEX, Framingham, MA, USA) was applied in viewing and processing raw data. BioPharmaView 3.0 (SCIEX, Framingham, MA, USA) was used in the multiple attributes monitoring (MAM) analysis of intact protein and peptide mapping.

## 5. Conclusions

Molecular heterogeneity was ubiquitous in recombinant proteins for post-translational modification and chemical reactions during expression, purification, and long-term storage. Identifying these modifications is an essential element of quality control of therapeutic recombinant proteins and offers critical quality attribute data to document and control process stability. Our study detected IFNα2b variants and site-specific modifications in seven therapeutic IFNα2b preparations from different manufacturers, suggesting that the level of oxidation and deamidation were vulnerable to many factors, which were hard to control, and the constitution of N-terminus and disulfide bonds was relatively stable between different batches, which may be a valid indicator for batch consistency. These findings provide a valid reference for the stability evaluation of the production process and final products.

## Figures and Tables

**Table 1:** Area percentages of peaks based on UV signal.

Sample No.	Peak 1 (%)	Peak 2 (%)	Peak 3 (%)	Peak 4 (%)	Peak 5 (%)
1E	/	/	99.31 ± 0.33	0.31 ± 0.003	0.38 ± 0.33
2E	/	/	100.00	/	/
3E	0.51 ± 0.02	/	99.49 ± 0.02	/	/
4E	/		74.89 ± 0.05	22.18 ± 0.02	1.89 ± 0.07
5E	0.19 ± 0.002	/	99.81 ± 0.002	/	/
6P	/	/	65.97 ± 0.06	13.67 ± 0.02	20.36 ± 0.06
7Y	/	0.74 ± 0.01	99.27 ± 0.004	/	/

**Table 2:** Identification of IFNα2b variants.

IFNα2b Variants	Retention Time (min)	Shift of Average MW (Da)	Theoretical Average MW (Da)	Measured Average MW (Da)	Error (Da)
Oxi	~14.2	+16.00	19,280.91	19,280.68	−0.23
		+32.00	19,296.91	19,296.50	−0.41
dOx	~16.0	−16.00	19,248.16	19,248.66	0.5
Native *	~16.4	/	19,264.91	19,264.19	−0.72
+Met		+131.20	19,396.11	19,395.33	−0.78
ACE	~17.2	+42.04	19,306.95	19,306.49	−0.46

* The amino acid sequence of native IFNα2b is from Chinese Pharmacopoeia, 2020 version.

**Table 3:** Deconvoluted area of oxidized IFNα2b in sample 4E and 6P.

IFNα2b Variant	Sample 4E				Sample 6P			
	1	2	3	Average	1	2	3	Average
IFNα2b + M, Oxidation-1	8690.8	8628.6	8580.7	8580.7	3604.8	3567.6	3609.5	3594.0
IFNα2b + M	24,271.3	22,641.7	22,905.7	22,905.7	23,047.2	21,887.2	23,409.9	22,781.4
IFNα2b, Oxidation-2	0.0	0.0	0.0	0.0	897.2	864.6	888.4	883.4
IFNα2b, Oxidation-1	4722.9	4960.8	4913.6	4913.6	1599.2	1727.5	1717.8	1681.5
IFNα2b	18,739.6	19,652.7	19,395.4	19,395.4	12,413.0	12,756.8	13,133.5	12,767.8
Oxidation in IFNα2b +M (%)	26.4	27.6	27.9	27.3	14.0	13.4	13.6	13.5
Oxidation in IFNα2b (%)	20.1	20.2	20.3	20.2	20.3	19.8	20.1	20.1
Oxidation in total IFNα2b (%)	23.8	24.3	24.5	24.2	15.1	14.5	14.8	14.7

## Supplementary Materials

The following are available online at https://www.mdpi.com/1420-3049/25/17/3965/s1, Figure S1: Non-deconvoluted mass spectra of IFNα2b variants.
