# Supplementary material for: Analysis of Molecular Heterogeneity in Therapeutic IFNα2b from Different Manufacturers by LC/Q-TOF
Source: Molecules. 2020 Aug 31;25(17):3965. doi: 10.3390/molecules25173965 (PMC7504738; doi:10.3390/molecules25173965)
Supplement: Supplementary file 1 [file molecules-25-03965-s001.pdf]

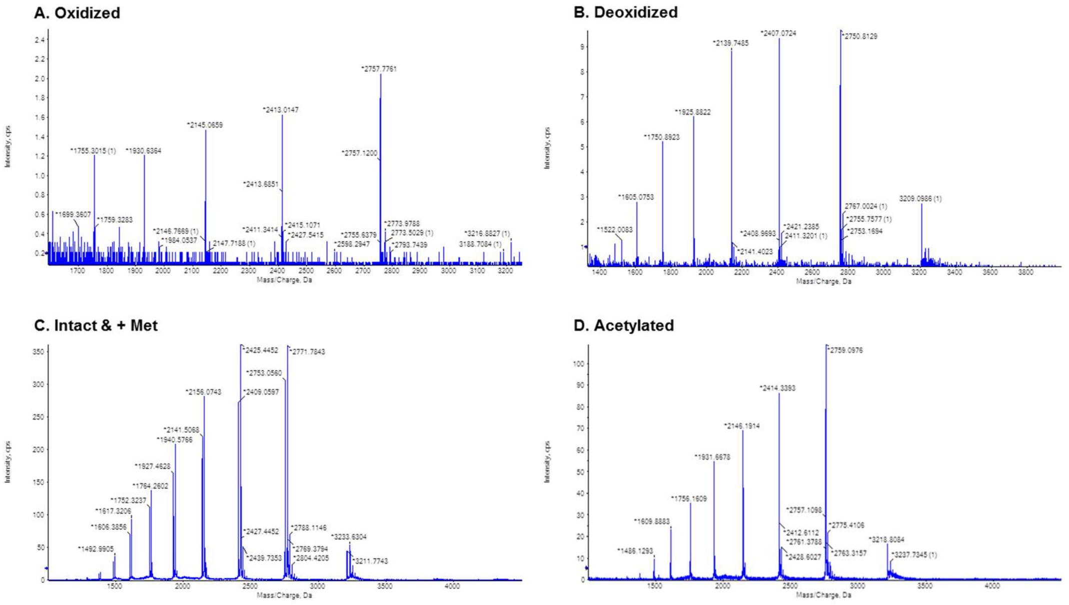

Figure S1**.** Non-deconvoluted mass spectra of IFNα2b variants. A. Spectrum from 14.095 to 14.420 min of sample 6P. B. Spectrum from 15.927 to 16.098 min of sample 7Y. C. Spectrum from 16.509 to 16.920 min of sample 6P. D. Spectrum from 17.246 to 17.588 min of sample 6P.
